# SysNatMed: rational natural medicine discovery by systems genetics

**DOI:** 10.3389/fphar.2025.1496061

**Published:** 2025-03-03

**Authors:** Chang-Qing Ye, Jie Leng, Meng-Yuan Jin, Yuan-Dong Meng, Zi-Yi Zhao, Fan-Xing Meng, Xuan Xu, Sha-Sha Fan, Hong-Bin Luo, Xiang-Yu Meng

**Affiliations:** ^1^ Hubei Key Laboratory of Biological Resources Protection and Utilization, Hubei Minzu University, Enshi, China; ^2^ Xiangyang Hospital of Traditional Chinese Medicine and Xiangyang Institute of Traditional Chinese Medicine, Xiangyang, China; ^3^ Health Science Center, Hubei Minzu University, Enshi, China; ^4^ School of Life Sciences, Anhui Medical University, Hefei, China; ^5^ Hubei Provincial Clinical Medical Research Center for Nephropathy, Hubei Minzu University, Enshi, China

**Keywords:** natural medicine, rational drug discovery, systems genetics, polypharmacology, complex disease

## Abstract

**Background:**

Although acknowledged as an important complement to modern medicine, the utility of natural medicine (NM) remains under-exploited. We aimed to develop a novel data-driven approach for natural medicine discovery.

**Methods:**

GWAS summary statistics of disease (Alzheimer’s disease, i.e., AD, for the case study) and quantitative trait loci were collected from public sources. The ranking of disease-gene associations was established using summary-based Mendelian randomization. The comprehensive hierarchical relationships among ingredients, natural products, and target genes were compiled from the BATMAN-TCM v2.0 database. Based on the ranking of disease-gene associations and the comprehensive hierarchical relationships among ingredients, natural products, and target genes, we prioritized NM ingredients as potential candidates for AD management and examined the efficacy for AD prevention using rat AD models.

**Results:**

We developed a non-trivial transparent data-driven framework for systems genetics-based NM discovery. Among the 139 prioritized candidates for AD management, we demonstrated the efficacy of *Dang Gui* (*Angelicae Sinensis Radix*, ASR) and *Dang Shen* (*Codonopsis Pilosula*, CP) for AD prevention using rat models. Mechanistically, we showed that ASR may prevent AD-related damage through protection of neural cells, as well as inhibition of microglia, angiogenesis, inflammation, and extracellular matrices.

**Conclusion:**

Our method holds potential for the development of new strategies of complementary medicine for disease treatment and prevention, especially for complex conditions involving a number of genes.

## Graphical Abstract

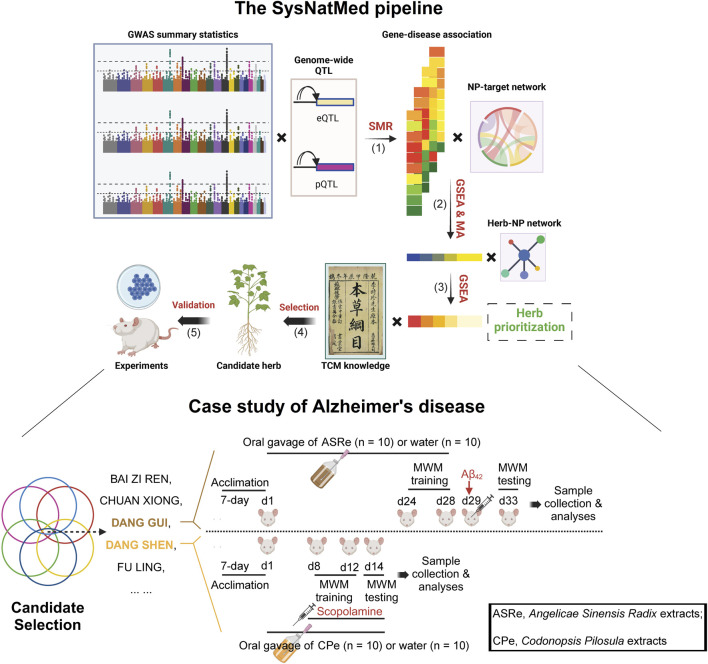

## 1 Introduction

As the population ages, the burden of disease continues increasing, particularly those disorders with complicated and unelucidated aetiology, such as neurodegenerative disorders, autoimmune diseases, and cancer. These complex diseases normally involve multiple contributing genetic and environmental factors, and present diverse manifestation phenotypes. Although advancesin modern medicine brings new insights for the management of complex diseases, the lack of effective strategy for prevention, detection, and treatment remains an urgent unmet need.

Accompanying human history, natural medicine uses natural resources for healthcare. It represents a crucial component of human civilization across diverse cultures, with the traditional Chinese medicine (TCM) as a living example of ongoing impact. Natural medicine demonstrates effectiveness in practice and gave birth to a number of key discoveries in modern medication, such as aspirin and artemisinin. While mechanistic understanding of natural medicine remains poor, a holistic polypharmocology perspective has been widely recognized. Different from modern medicine that emphasis on single target therapeutics, traditional nature medicine normally adopts a polypharmacology perspective that modulates multiple pathways in one shot. This polypharmacological strategy may particularly fit in the context of management of complex disease, including disease prevention which is a key concept of TCM. However, traditional practice of nature medicine usually relies on accumulated historical experience and needs to integrate novel rational strategies based on scientific evidence, and increasing efforts have been made in this direction. Of note, a recent piece of work by Dr. Albert-László Barabási’s team proposed a systems medicine framework that theorizes the scientific basis of TCM, laying a foundation of rational herb predication for various health conditions (Gan et al., 2023).

Following previous works, we propose in this study the SysNatMed, a systems genetics approach that identifies natural medicine candidates for human diseases, by integrating gene-disease connectivity and herb-natural product (NP)-target networks. Briefly, the SysNatMed approach first computationally estimates gene-disease associations using proteome-wide and transcriptome-wide Mendelian randomization analysis, which is an established method that has been successfully applied in drug discovery and repurposing, while other methods such as transcriptome-wide association study (TWAS) could be alternatives when appropriate (Mai et al., 2023; Gaziano et al., 2021). After further processing steps, the computed gene-disease associations were jointly analyzed with herb-NP-target networks for prioritization of NPs and/or herbs, before filtering or integration with traditional medicine expertise and functional experiments for disease-specific and even personalized drug discovery.

## 2 Materials and methods

The detailed materials and methods of the SysNatMed approach, especially those related with the case study of Alzheimer’s disease (AD), are described as below.

### 2.1 Collection of GWAS data for Alzheimer’s disease

We searched the IEU OpenGWAS database for datasets of GWAS summary statistics of Alzheimer’s disease (AD), using the search term ‘alzheimer’ (Elsworth et al., 2020). Datasets with family history of Alzheimer’s disease as phenotype of interest were not considered. Three datasets of European ancestry, the ieu-b-5067 (n = 488,285; #SNPs = 12,321,875), the ebi-a-GCST90027158 (n = 487,511; #SNPs = 20,921,626), and the finn-b-G6_ALZHEIMER (n = 218,792; #SNPs = 16,380,466), were finally included (Larsson et al., 2022; Kurki et al., 2023).

### 2.2 Collection of GWAS data for QTLs

GWAS summary statistics of eQTLs were collected from two sources, the eQTLGen and the GTEx v8 (Võsa et al., 2021; Consortium et al., 2020). For eQTLGen, data for significant *cis*-eQTLs were downloaded. For GTEx v8, the multi-tissue Metasoft results were downloaded. GWAS summary statistics of pQTLs were collected from two sources, the UKB-PPP and the deCODE pQTL (Sun et al., 2023; Ferkingstad et al., 2021). For QTL data based on the hg38 reference genome, the chromosome coordinates of the SNPs were lifted-over to that of the hg19 reference genome before subsequent analyses.

### 2.3 Compilation of NP-target and herb-NP relationships

We downloaded the interactions between traditional Chinese medicine (TCM) ingredients (mostly TCM herbs), chemical compounds (mostly natural products), and target genes of the compounds, from the BATMAN-TCM v2.0 database (Kong et al., 2024). A total of 2,336,340 literature-derived and computationally-predicted interactions for ∼8400 TCM ingredients, ∼40,000 chemical compounds, and ∼10,000 target genes were used for subsequent bioinformatics analysis.

### 2.4 SMR-based prioritization of genes

Protein or mRNA expression of genes were considered exposure for Mendelian randomization and QTL summary statistics were used for the construction of genetic instruments. We accordingly constructed transcriptome-wide and proteome-wide genetic instruments. Briefly, for each gene, the corresponding significant QTL SNPs (FDR < 0.05) were extracted and LD-clumped using default parameters and European population data. Summary-data-based Mendelian randomization (SMR) was performed for each of the AD and QTL dataset pairs (a total of 3*4 = 12 analyses) (Zhu et al., 2016). Briefly, for the instrumental SNPs in a given QTL dataset, the corresponding AD summary statistics were extracted from the IEU OpenGWAS database using the *extract_outcome_data* function with a ‘proxies = T’ argument of the TwoSampleMR R package. The QTL exposure data and the corresponding AD outcome data were merged and harmonized using the *harmonise_data* function of the TwoSampleMR package with default parameters, and the resulting data matrix was then subjected to SMR analysis using the *SMR* function of the SMRinR package with default parameters. The -log10 transformed nominal *P*-values of SMR analysis results were used as the ranking metric for association between AD and genes.

### 2.5 Prioritization of NPs and herbs for AD

First, for NP prioritization, the ranks of genes by SMR-based prioritization were used for gene-set enrichment analyses (GSEA) (Subramanian et al., 2005), which in our AD case study each NP was considered as a set and its target genes as the items in this specific set, extracted from the NP-target interactions. GSEA were performed using the *fgsea* function of the fgsea R package with default parameters except ‘eps = 0’ and ‘scoreType = pos’. In case where multiple GSEA results were available, such as the case of our AD study, the *P*-vaules of the NPs were further pooled using meta-analysis by Stouffer’s method, which combines the p-values using the sum of z (Mullen, 1982). The final meta-analyzed *P*-values of each NP were -log10 transformed and used as the prioritizing metric for the NPs.

Next, the ranks of NPs were used for the final GSEA, which in our AD case study each TCM ingredient (mostly herbs) was considered as a set and its associated chemical compounds (mostly natural products) as the items in this specific set, extracted from the herb-NP interactions. GSEA were performed same as above described. The *P*-values were adjusted for multiple comparisons using the Bonferroni corrections, and the herbs with an adjusted *P*-value < 0.05 were considered the potential prioritized herb candidates for AD management.

### 2.6 Collection of TCM functions and effects

We collected the TCM knowledge on therapeutic functions and effects of the prioritized herb candidates for AD management, using a few trusted sources of information, including the *Encyclopedia of Chinese Materia Medica*, the *Compendium of Materia Medica*, and the *Chinese Materia Medica* (see Supplementary Table S1 for titles in Chinese).

### 2.7 Collection of TCM patents for AD and associated herbs

We searched the Wanfang Patent Database (https://c.wanfangdata.com.cn/patent), a comprehensive database of Chinese patents, for identification of TCM patents for AD, using search terms that are linguistic equivalents in Chinese to that of [“Alzheimer’s disease” AND “Chinese herbal medicine”] (see Supplementary Table S1 for search terms in Chinese). The patents yielded by the search were screened for eligibility, and those described clear usage of TCM ingredients for AD, either in formula or single, were considered. A total of 317 patents containing 521 non-duplicated TCM ingredients were used for further analyses.

### 2.8 Animals for experiments

Male Sprague-Dawley rats were purchased from the Liaoning Changsheng Biotechnology Co., Ltd. (Benxi, Liaoning, China; License for experiment animals: SCXK (辽) 2020–0001; #QC pass: 210726220100765686 and 210726230101434841). 30 rats of 2–3 months old and 200 ± 20 g weight were included for experiments for Dang Gui (*Angelicae Sinensis Radix*, ASR) and Dang Shen (*Codonopsis Pilosula*, CP), respectively. The animals were fed in a standardized animal facility equipped with individual ventilation cages, with ambient temperature kept 25°C ± 3°C and feeding and water-drinking *ad libitum*. The shipped animals were subjected to 1-week acclimation before experiments. The animal study protocols were approved by the institutional review board of Hubei Minzu University [No. 2023043 and No. 2023069] and conforms with ethnic requirements. For experiments concerning ASR, the 30 rats were randomly allocated to the Control (no treatment), Model (Aβ_42_), and ASR (ASR extracts + Aβ_42_) groups with 10 animals for each group. For experiments concerning CP, the 30 rats were randomly assigned to the Control, Model (scopolamine), and CP (CP extracts + scopolamine) groups with 10 animals for each group.

### 2.9 Materials and devices

The herbs of ASR and CP were purchased from cultivators located in Enshi Tujia and Miao Autonomous Prefecture, and the authenticity and quality was checked and endorsed by Dr. Hou-Cong Li, expert in herb medicine of Enshi region, affiliated to the Lab of Chinese Medicinal Herbs, Health Science Center, Hubei Minzu University. The Nissl staining solution (lot. 082521220609) was purchased from Beyotime Biotechnology Co., Ltd. (Shanghai, China). The Aβ_42_ (lot. 04010011827) was purchased from Yaoqiang Biotechnology Co., Ltd. (Shanghai, China). The scopolamine hydrobromide was purchased from MedChemExpress (lot. 252414). The Morris Water Maze system was manufactured by Institue of Materia Medica, Chinese Academy of Medical Science (Shanghai, China). The vibrating microtome was manufactured by Leica Biosystems (Shanghai, China).

### 2.10 Treatment preparation and administration

Decoctions with a concentration of 0.226 g/mL and 0.141 g/mL were prepared for ASR and CP, respectively. For preparation of Aβ_42_, we resolved 1 mg Aβ_42_ to 20μL DMSO, then diluted the solution with double distilled water to 500ul to have a final concentration of 2 μg/μL. The Aβ_42_ preparation was incubated in dark at 37°C for 7 days to form a fibroid aged Aβ_42_ formulation. Preparation procedures of Aβ_42_ was under strict sterile conditions. Prevention using ASR or CP extracts were administered for the ASR and CP prevention groups of rats (n = 10 each) through oral gavage at dosage of 2.26 g/kg and 1.41 g/kg for 28 and 14 days, respectively.

### 2.11 AD modeling

#### 2.11.1 Aβ_42_-based AD modeling

Rats were weighed and then administered 20% urethane anesthesia via intraperitoneal injection at a dose of approximately 0.5 mL/100 g. After full anesthesia was achieved, the scalp on the top of the rats’ heads was prepared. The rats’ tongues were gently pulled out using one end of a cotton swab, and the rats were secured on a stereotaxic frame. The surgical area was disinfected with iodine. A neat incision approximately 1–2 cm long was made along the midline using scissors. The incision was cleaned with a cotton swab dipped in 3% hydrogen peroxide, and the “sagittal suture” was located. Referring to the stereotaxic atlas of the rat brain, holes were drilled into the skull at 0.8 mm posterior to the bregma and 1.5 mm lateral on each side. Using a 5 µL Hamilton syringe, 5 µL of Aβ_42_ solution was drawn up. The syringe was inserted vertically and slowly to a depth of 3.5 mm into both lateral ventricles. The Aβ_42_ solution was injected slowly over about 5 min. The needle was left in place for an additional 5 min after injection, then slowly withdrawn. The same procedure was repeated on the opposite side. The incision was then sutured and disinfected with iodine. The rats were then placed in clean, padded cages in a lateral position and kept in a warm environment until they recovered.

#### 2.11.2 Scopolamine-based AD modeling

A 0.4 mg/mL solution of scopolamine hydrobromide was prepared in sterile saline. Rats were weighed and scopolamine hydrobromide solution was administered intraperitoneally at a dosage of 0.5 mL per 100 g of body weight. The administration of scopolamine was repeated daily for a consecutive period of 14 days.

### 2.12 Morris water maze experiments

A Morris water maze (MWM), composed of a circular pool, a black platform, and a video capture system, was used. Various distinct icons were affixed to the walls of the room housing the maze, which was both soundproof and dimly lit to minimize external influences on the subjects. Prior to the commencement of training, rats were placed in the room to acclimate overnight. The black platform was positioned in the third quadrant of the pool, and the pool was filled with enough water to submerge the platform by approximately 1 cm. The water was dyed black with ink to obscure the platform, and the temperature within the pool was maintained at 24°C ± 1°C.

The MWM experiments consisted of two parts: localization training and testing. During training, each rat was sequentially introduced into the water from the same location in the first, second, and fourth quadrants. The swimming trajectories and related data of the rats were recorded by the video capture system for 60 s. If a rat located the platform within 60 s, it was allowed to remain on the platform for 5 s to familiarize itself with the surroundings. If the platform was not found within 60 s, the rat was guided to the platform and allowed to stay for 15 s to help it memorize the location of the platform.

Following the accomplishment of Alzheimer’s disease modeling and a recovery period where the rats resumed normal activity within 24 h, the water maze test was conducted without the platform, and the swimming data after the rats were introduced into the first quadrant were recorded. Specifically, for ASR and CP, we did as follows (Gan et al., 2023): For the ASR and related non-treatment Model groups, the rats began MWM training on the 23rd day after the start of oral gavage of ASR or water, for five consecutive days. On the 28th day, after anesthesia, 5 µL of a 2 μg/μL Aβ_42_ solution was injected at specific cranial coordinates. After recovery of activity, within 24 h, the MWM test was performed, and the swimming routes and the number of times that the rats crossed the platform were recorded (Mai et al., 2023); For the CP and related non-treatment Model groups, rats received an intraperitoneal injection of 0.4 mg/mL scopolamine hydrobromide (0.5 mL per 100 g body weight) starting on the 8th day after the commencement of oral gavage of CP or water, daily for 7 days. MWM training began on day 8 and continued for 5 days. The MWM testing was performed on day 14. The swimming routes and the number of times that the rats crossed the platform were recorded.

### 2.13 Sample preparation and analysis

#### 2.13.1 Nissl staining

Rats (n = 3 for each group) were anesthetized and subsequently perfused transcardially with physiological saline followed by a 4% paraformaldehyde solution. The brains were then carefully removed and further fixed by immersion in 4% paraformaldehyde solution and stored at 4°C. The brains were sectioned using a vibrating blade microtome to produce continuous coronal slices of 25 µm thickness. These brain slices were transferred to glass slides and allowed to air-dry naturally. Nissl staining solution was dropped onto the dried brain slices. The slides were then placed in a 37°C incubator for 5 min. After incubation, excess staining solution was removed, and the slices were rinsed twice with ddH_2_O for 10 s each, followed by air drying. The air-dried brain slices were immersed in 95% alcohol for dehydration, each immersion lasting for 5 min and repeated twice. This was followed by clearing in xylene for 2 min, also repeated twice. After clearing, an adequate amount of neutral balsam was applied to the slices. Once the balsam had dried, the slides were ready for microscopic examination.

#### 2.13.2 RNA-seq

Hippocampus were sampled from the AD model rats with and without ASR prevention (n = 3 each). Total RNAs were extracted and subjected to quality control before library preparation, which was then quantified with Qubit 2.0, assessed with Agilent 2000 for insert size, and then quantified with qPCR. The quality-controlled library was sequenced using 150bp pair end reads on the Illumina NovaSeq platform. The fastq files were trimmed for adapter sequences and quality, and then mapped to the rat reference genome. Raw read counts were then calculated before differential expression analysis with DESeq2 R package. Genes were ranked by the log fold-change values of differential expression between the ASR-treated and non-treated AD rats, followed by GSEA concerning signatures (gene sets) of human aging brain, human AD brain, human brain cell markers, and AD-related functional pathways.

#### 2.13.3 Statistical analysis

Continuous data were visualized use bar, dot and/or box plots. Non-parametric Kruskal–Wallis test and Wilcoxon test was used for statistical comparisons.

#### 2.14 Generative AI technologies

GPT-4-Turbo was used for writing aid in part of the Methods sections.

## 3 Results and discussion

**FIGURE 1:**
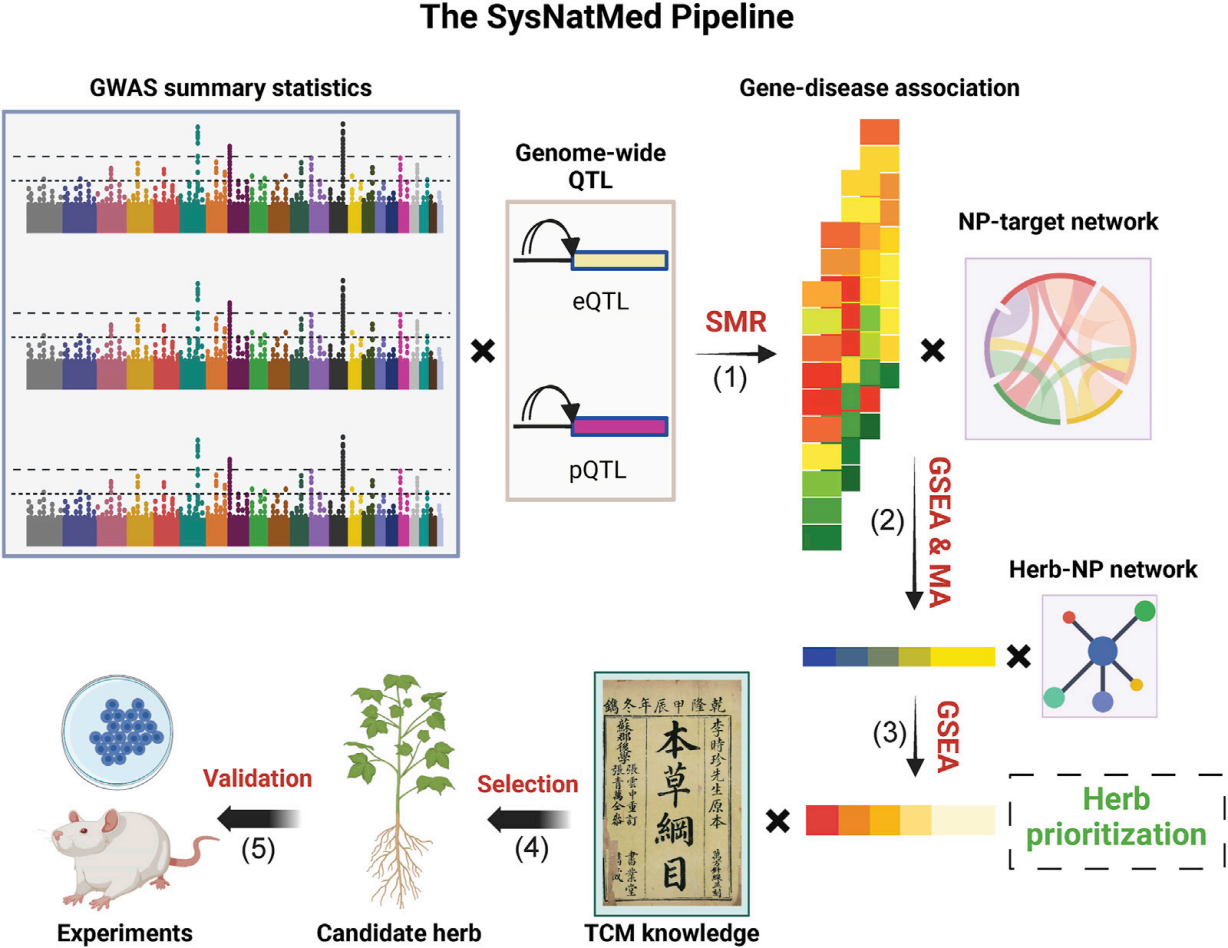
Schematic illustration of the SysNatMed. GSEA, gene-set enrichment analysis; MA, meta-analysis; NP, natural product; QTL, quantitative trait loci; SMR, summary-based Mendelian randomization; TCM, traditional Chinese medicine. The major steps include: (1) SMR-based gene-disease association estimation; (2) Prioritization of NPs, in terms of the modulation potential of the disease/condition of interest, by joint analysis of gene-disease association and NP-target relationships through GSEA or subsequent consensus ranking by MA (3) Prioritization of natural medicine ingredients (e.g., herbs); by GSEA based on NP rankings, to propose candidates; (4) Refinement of candidates by joint consideration of systems genetics rankings and knowledge of natural medicine (e.g., TCM principles); (5) Experimental validation using preclinical models before clinical application.

The SysNatMed is a non-trivial transparent data-driven framework. As shown in Figure 1, it constitutes four major steps (Gan et al., 2023): GWAS data (e.g., summary statistics) for a given disease of interest and molecular quantitative trait loci (e.g., eQTLs and pQTLs) are used for estimation of gene-disease associations by summary-based Mendelian randomization (SMR), a genetics method widely used for inference of causal relationships between genes and phenotypes (Gaziano et al., 2021). Mai et al. (2023) Taking into account the rank of genes by association with the disease and the NP-target relationships, gene-set enrichment analyses (GSEA) are performed to prioritize the NPs in terms of the possible modulation effect on the disease of interest; and in case where multiple GWAS and/or QTL data are analyzed, the individual NP prioritization results are aggregated to a consensus ranking by meta-analysis of *P*-values (Gaziano et al., 2021); Further GSEA are performed based on the consensus rank of NPs and the herb-NP relationships to prioritize the herbs in terms of the possible utility for the disease of interest (Elsworth et al., 2020); Top candidate herbs are screened and refined with prior knowledge (e.g., TCM classics, patents and/or clinical observations) (Larsson et al., 2022); Functional validation in model systems before clinical trials in human subjects.

**FIGURE 2:**
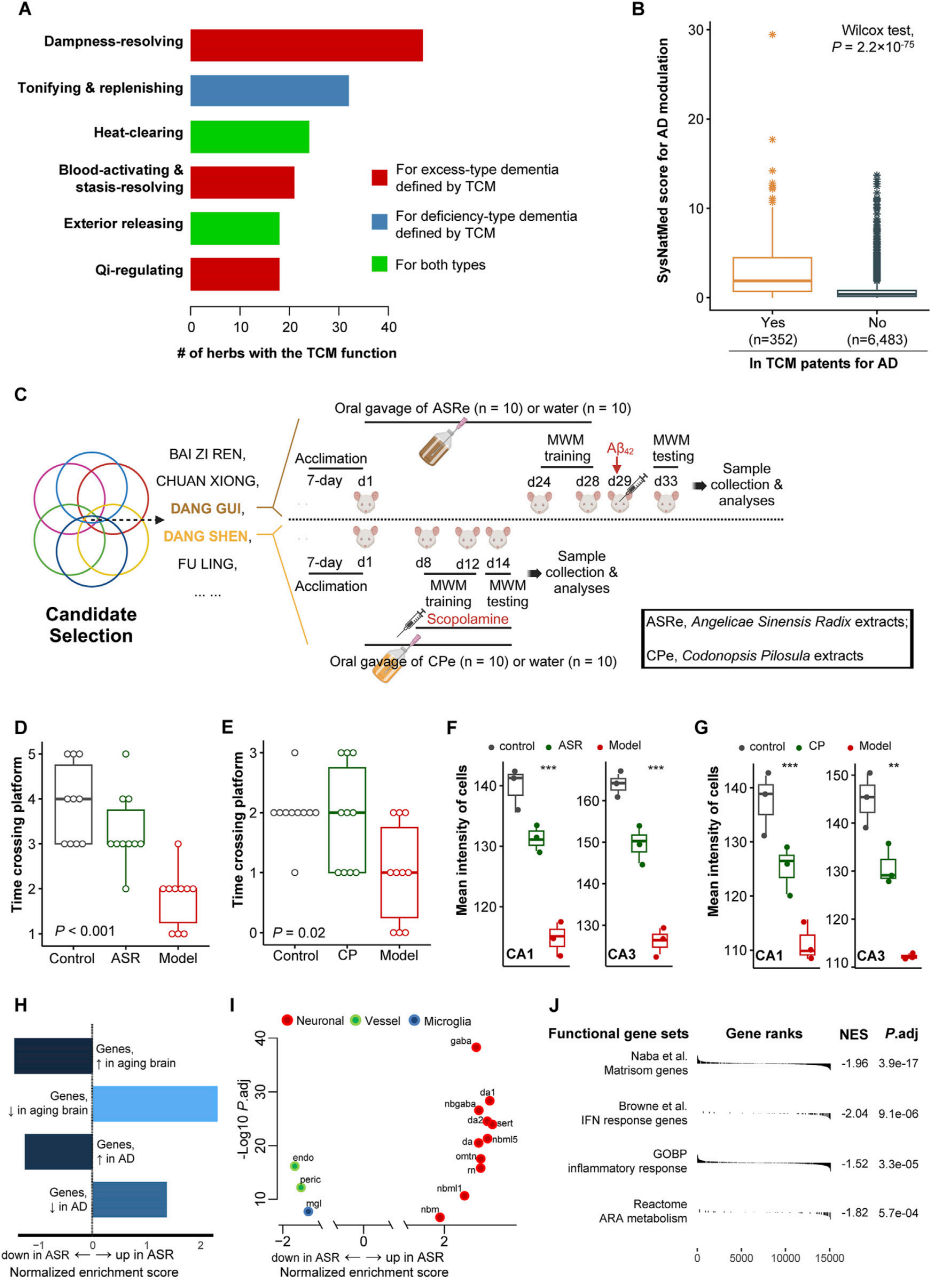
Case study of applying SysNatMed to AD. **(A)** Therapeutic effects targeting AD defined by traditional Chinese medicine (TCM) among the 139 herbs prioritized by SysNatMed for AD. **(B)** The SysNatMed score for AD modulation between ingredients included or not in patents of TCM for AD. The higher the score, the higher confidence suggested by SysNatMed that the herb will have an impact on AD. **(C)** Workflow of candidate herb selection and subsequent experiments using AD rat models. MWM, Morris water maze. A 5-day place navigation training plus a final set of spatial probe trails for testing of cognitive performance. **(D–E)** Bouts crossing platform in spatial probe trials. ASR, model animals underwent prevention with extracts of *Angelicae Sinensis Radix*; CP, model animals underwent prevention with extracts of *Codonopsis Pilosula*; Model, model animals without herb treatment; Control, animals free of AD modeling and herb treatment. **(F–G)** Mean intensity of cells in CA1 and CA3 regions of hippocampus across groups of animals, as revealed by Nissl staining. **(H–J)** Gene-set enrichment analysis following differential gene expression of ASR versus model groups, using signatures of aging and AD brain **(H)**, brain cell markers **(I)**, and AD-related functional pathways **(J)**. A positive normalized enrichment score suggests higher expression level of the signature genes in hippocampus of AD-model rats treated with ASR. omtn, oculomotor and trochlear nucleus; sert, serotonergic; nbm, medial neuroblast; da, dopaminergic neurons; rn, red nucleus; gaba, GABAergic neurons; nbml, mediolateral neuroblasts; mgl, microglia; endo, endothelial cells; peric, pericytes.

We examined the potential applicability of SysNatMed in a case study of Alzheimer’s disease. AD is a devastating and irreversible neurodegenerative disorder that makes large and yet increasing burden of disease. Despite recent progress in understanding the pathophysiology of AD, current therapeutic and preventive options remain limited. While certain medications can help relieve the symptoms, there are no cures capable of reversing the underlying pathology, and strategies for effectively prevention are yet to be established (Xu et al., 2024). Natural medicine represents a potential alternative for AD management. We applied the SysNatMed to AD, using a collection of GWAS summary statistics of AD from the IEU OpenGWAS database (ebi-a-GCST90027158, finn-b-G6_ALZHEIMER, and ieu-b-5067) and QTLs from the eQTLgen, GTEx, deCODE, and UKB-PPP sources, as well as the NP-target and herb-NP relationships curated from the BATMAN-TCM v2 database (Kong et al., 2024). Genome-wide associations between genes and AD were calculated using SMR, followed by prioritization of NPs and further TCM herb ingredients (Supplementary Tables S2, S3). Among the 8,399 herbs analyzed, a total of 139 prioritized herbs passed the statistical significance thresholds (Bonferroni-corrected *P* < 0.05; Table 1). Reviewing TCM literature, we noted that the TCM functions of these herbs were in well concord with the theoretic principles in TCM management of senile dementia syndromes. Of note, the TCM effect of ‘dampness/phlegm-resolving’, which is crucial for the management of the ‘excess-type dementia’, was the most frequent among these herbs (47/139, 33.8%), followed by the TCM effect of ‘tonifying and replenishing’ (32/139, 23.0%), which is key for the management of the ‘deficiency-type dementia’ (Figure 2A). Additionally, the herbs included in TCM patents for AD therapeutics (n = 521, extracted from 317 patents) showed a systematically higher score in terms of the potential in modulating AD, reiterating the validity of our approach from a TCM clinical perspective (Wilcox rank-sum test, *P* = 2.2 × 10^−75^; Figure 2B).

**TABLE 1:** Traditional Chinese Medicine ingridients prioritized by SysNatMed for potential modulation on AD (Bonferroni adjusted *P* < 0.05).

Ingridients	NES	P-value	P.fdr	P.Bonferroni	# of NP targets
QIANG HUO	1.54	3.49E-30	2.39E-26	2.39E-26	386
MU GUA	1.55	2.04E-18	6.98E-15	1.40E-14	199
BAI ZI REN	1.68	6.42E-15	1.46E-11	4.39E-11	84
CHUAN SHE GAN	1.57	1.94E-14	2.84E-11	1.33E-10	138
BIN LANG	1.74	2.08E-14	2.84E-11	1.42E-10	63
XU CHANG QING	1.68	5.57E-14	6.35E-11	3.81E-10	81
NAN GUA ZI	1.77	9.93E-14	9.69E-11	6.79E-10	52
**DANG GUI (Angelicae Sinensis Radix)**	**1.40**	**1.47E-13**	**1.25E-10**	**1.00E-09**	**307**
CHANG CHUN HUA	1.55	1.81E-13	1.38E-10	1.24E-09	137
HE ZI	1.62	2.54E-13	1.74E-10	1.74E-09	93
MU HU DIE	1.56	3.42E-13	2.13E-10	2.34E-09	131
**DANG SHEN (Codonopsis Pilosula)**	**1.40**	**4.60E-13**	**2.53E-10**	**3.14E-09**	**285**
NIU BANG ZI	1.46	5.04E-13	2.53E-10	3.45E-09	201
ROU GUI	1.46	5.19E-13	2.53E-10	3.55E-09	202
MA CHI XIAN	1.56	6.81E-13	3.10E-10	4.65E-09	115
SHI JUN ZI	1.62	1.83E-12	7.81E-10	1.25E-08	88
TIAN KUI ZI	1.65	7.26E-12	2.80E-09	4.97E-08	75
GOU QI ZI	1.36	7.38E-12	2.80E-09	5.05E-08	306
BING LANG	1.62	8.34E-12	2.97E-09	5.70E-08	86
GAN DI HUANG	1.49	8.71E-12	2.97E-09	5.95E-08	157
BA DOU	1.58	1.09E-11	3.54E-09	7.43E-08	104
SHE CHUANG ZI	1.47	1.21E-11	3.75E-09	8.25E-08	164
KU XING REN	1.50	1.32E-11	3.91E-09	8.99E-08	144
ZI SU ZI	1.51	1.60E-11	4.57E-09	1.10E-07	140
CHU SHI ZI	1.53	2.11E-11	5.77E-09	1.44E-07	127
CHUAN WU	1.46	3.61E-11	9.27E-09	2.47E-07	169
HU JIAO	1.43	3.66E-11	9.27E-09	2.50E-07	202
CU LIU GUO	1.50	4.20E-11	1.02E-08	2.87E-07	132
LA JIAO	1.40	7.26E-11	1.71E-08	4.96E-07	233
TIAN NAN XING	1.50	7.85E-11	1.79E-08	5.36E-07	136
CANG ER ZI	1.44	8.34E-11	1.84E-08	5.70E-07	176
WU MEI	1.50	1.03E-10	2.20E-08	7.03E-07	124
YUAN SUI	1.36	1.07E-10	2.21E-08	7.30E-07	289
XIE BAI	1.55	1.22E-10	2.46E-08	8.37E-07	101
LING XIAN	1.63	1.44E-10	2.81E-08	9.84E-07	72
HONG HUA	1.38	1.77E-10	3.37E-08	1.21E-06	252
MAO ZHUA CAO	1.61	4.08E-10	7.53E-08	2.79E-06	73
LU HUI	1.46	4.31E-10	7.76E-08	2.95E-06	147
XIAO HUI XIANG	1.61	7.95E-10	1.39E-07	5.44E-06	66
GUANG ZAO	1.44	8.19E-10	1.39E-07	5.60E-06	158
MA BIAN CAO	1.43	8.35E-10	1.39E-07	5.71E-06	167
CHAI HU	1.31	9.62E-10	1.57E-07	6.58E-06	366
DI FU ZI	1.65	1.04E-09	1.66E-07	7.12E-06	54
GAN LAN	1.64	1.52E-09	2.36E-07	1.04E-05	56
FU LING	1.43	1.90E-09	2.89E-07	1.30E-05	155
HA MA YOU	1.69	2.54E-09	3.78E-07	1.74E-05	46
HUO MA REN	1.41	2.77E-09	4.02E-07	1.89E-05	178
WU ZHU YU	1.36	2.86E-09	4.07E-07	1.96E-05	227
FENG FANG	1.59	2.93E-09	4.08E-07	2.00E-05	65
CHUAN XIONG	1.31	3.21E-09	4.39E-07	2.20E-05	343
BAI WEI	1.62	3.31E-09	4.44E-07	2.26E-05	60
MA HUANG	1.29	3.70E-09	4.85E-07	2.53E-05	417
SHUI FEI JI	1.55	3.76E-09	4.85E-07	2.57E-05	82
BA JI TIAN	1.42	4.92E-09	6.22E-07	3.36E-05	160
WEI LING XIAN	1.44	5.04E-09	6.27E-07	3.45E-05	151
BAN LAN GEN	1.35	6.91E-09	8.43E-07	4.72E-05	219
WU HUA GUO	1.49	7.30E-09	8.75E-07	4.99E-05	107
BIE JIA	1.62	8.46E-09	9.97E-07	5.78E-05	57
SHA REN	1.31	8.72E-09	1.01E-06	5.96E-05	310
YU MI XU	1.52	9.36E-09	1.07E-06	6.40E-05	88
BAI BU	1.43	9.62E-09	1.08E-06	6.58E-05	151
GUA LOU	1.44	1.10E-08	1.20E-06	7.55E-05	142
JU HE	1.64	1.10E-08	1.20E-06	7.55E-05	50
SHA JI	1.37	1.34E-08	1.43E-06	9.14E-05	195
MAN JING ZI	1.37	1.52E-08	1.60E-06	1.04E-04	192
TIAN WEN CAO	1.79	1.60E-08	1.65E-06	1.09E-04	15
XI GUA	1.54	1.93E-08	1.97E-06	1.32E-04	76
CHUAN LIAN ZI	1.46	2.17E-08	2.17E-06	1.48E-04	112
WANG BU LIU XING	1.67	2.19E-08	2.17E-06	1.50E-04	40
JIU CAI ZI	1.65	2.35E-08	2.29E-06	1.61E-04	46
YIN JIAN	1.73	2.58E-08	2.49E-06	1.77E-04	30
FANG FENG	1.36	2.69E-08	2.55E-06	1.84E-04	205
PU TAO 2	1.37	3.05E-08	2.86E-06	2.09E-04	197
HUAN YU	1.78	3.63E-08	3.35E-06	2.48E-04	20
SANG SHEN	1.41	3.73E-08	3.40E-06	2.55E-04	148
GE HUA	1.34	4.30E-08	3.87E-06	2.94E-04	223
FENG LA	1.79	4.36E-08	3.87E-06	2.98E-04	13
BAI BIAN DOU	1.49	4.57E-08	4.00E-06	3.12E-04	93
DA ZAO	1.33	5.40E-08	4.67E-06	3.69E-04	242
XIONG GUO	1.79	6.66E-08	5.69E-06	4.55E-04	6
SHUANG BIAN GUA LOU	1.64	7.08E-08	5.97E-06	4.84E-04	46
CAO KOU REN	1.79	7.71E-08	6.42E-06	5.27E-04	6
XI HONG HUA	1.42	8.13E-08	6.69E-06	5.55E-04	135
YI YI REN	1.53	8.97E-08	7.30E-06	6.13E-04	78
SANG YE	1.28	1.09E-07	8.73E-06	7.42E-04	345
YIN CHEN HAO	1.39	1.13E-07	8.96E-06	7.71E-04	150
XI JIAO	1.61	1.21E-07	9.52E-06	8.28E-04	47
HAI MA	1.69	1.36E-07	1.04E-05	9.29E-04	29
KU LIAN ZI	1.50	1.36E-07	1.04E-05	9.29E-04	81
QIAN SHI	1.54	1.70E-07	1.29E-05	1.16E-03	63
SAN QI	1.27	1.84E-07	1.39E-05	1.26E-03	366
CHAO SU ZI	1.75	1.97E-07	1.46E-05	1.35E-03	21
QIAN JIN BA	1.48	2.18E-07	1.60E-05	1.49E-03	91
HEI DOU	1.36	2.82E-07	2.05E-05	1.93E-03	175
NIU DA LI	1.56	2.94E-07	2.12E-05	2.01E-03	59
PAO HE ZI	1.73	3.41E-07	2.43E-05	2.33E-03	24
JIANG HUANG	1.37	3.45E-07	2.43E-05	2.36E-03	159
BAI GUO	1.36	4.56E-07	3.14E-05	3.11E-03	163
FENG MI	1.44	4.56E-07	3.14E-05	3.11E-03	106
DONG KUI GUO	1.44	4.81E-07	3.29E-05	3.29E-03	106
SU ZI	1.74	5.49E-07	3.72E-05	3.75E-03	19
JING JIE	1.34	5.92E-07	3.97E-05	4.04E-03	179
BAI GUI BI	1.65	6.43E-07	4.27E-05	4.39E-03	36
WU HUAN ZI	1.62	6.60E-07	4.34E-05	4.51E-03	39
GUI BAN	1.65	6.94E-07	4.52E-05	4.74E-03	36
LUO HAN GUO	1.37	7.71E-07	4.97E-05	5.27E-03	142
SUAN ZAO	1.68	7.79E-07	4.98E-05	5.32E-03	25
TAO ZHI	1.50	8.22E-07	5.20E-05	5.62E-03	71
WU GONG	1.57	8.39E-07	5.26E-05	5.73E-03	48
SHENG JIANG	1.24	8.81E-07	5.48E-05	6.02E-03	406
HAI HONG DOU	1.69	9.16E-07	5.59E-05	6.26E-03	26
JI SHI TENG	1.35	9.16E-07	5.59E-05	6.26E-03	171
JIN YIN HUA	1.26	9.67E-07	5.85E-05	6.61E-03	333
CHUAN JIN PI	1.66	9.84E-07	5.85E-05	6.73E-03	33
MO SANG BI KE MEI DENG MU	1.78	9.84E-07	5.85E-05	6.73E-03	3
SHA YUAN ZI	1.38	1.09E-06	6.40E-05	7.43E-03	143
HAI JIN SHA	1.45	1.14E-06	6.65E-05	7.78E-03	92
WU YAO	1.35	1.55E-06	8.97E-05	1.06E-02	153
SHUI QIN	1.75	1.65E-06	9.48E-05	1.13E-02	8
MU JIN PI	1.69	1.91E-06	1.09E-04	1.30E-02	22
QIAO MAI	1.41	1.98E-06	1.12E-04	1.35E-02	106
MU MIAN HUA	1.31	2.08E-06	1.16E-04	1.42E-02	202
XIAN MAO	1.49	2.08E-06	1.16E-04	1.42E-02	66
BAI SU ZI	1.60	2.25E-06	1.24E-04	1.54E-02	41
XI HE LIU	1.37	2.63E-06	1.44E-04	1.80E-02	132
GUI ZHEN CAO	1.42	2.77E-06	1.49E-04	1.89E-02	90
LUO FU MU	1.63	2.77E-06	1.49E-04	1.89E-02	29
MA QIAN ZI	1.42	3.04E-06	1.63E-04	2.08E-02	94
XING REN	1.53	3.35E-06	1.78E-04	2.29E-02	51
HEI ZHI MA	1.39	3.49E-06	1.84E-04	2.39E-02	111
YUN NAN SHI ZI	1.64	3.84E-06	2.00E-04	2.62E-02	28
MU BIE ZI	1.63	4.04E-06	2.09E-04	2.76E-02	33
YU JIN	1.27	4.25E-06	2.18E-04	2.91E-02	267
GONG LAO MU	1.46	5.08E-06	2.59E-04	3.47E-02	78
XI YANG SHEN	1.29	5.15E-06	2.61E-04	3.52E-02	213
YANG JIAO MIAN	1.73	5.50E-06	2.76E-04	3.76E-02	14
YA MA ZI	1.54	5.56E-06	2.78E-04	3.80E-02	44
AN YE	1.59	5.70E-06	2.82E-04	3.90E-02	38
BIAN XU	1.40	5.98E-06	2.90E-04	4.09E-02	101
GUI ZHI	1.30	5.98E-06	2.90E-04	4.09E-02	215
ZI SU	1.22	5.98E-06	2.90E-04	4.09E-02	393
BAI ZHI	1.27	6.60E-06	3.18E-04	4.51E-02	259

The two ingredients in bold are the selected candidates used for validation in functional experiments.

We next performed functional validation for certain carefully selected candidate herbs. The following selection criteria were considered to identify the candidates: (a) Within the 139 herbs prioritized by SysNatMed; (b) Included in at least 10 patents of TCM for AD; (c) With at least one TCM effect useful for AD management; (d) Being medicinal and edible (i.e., medicine-food herb), for better safety and utility for prevention; (e) No known toxic effect; (f) Not in lists of wildlife protection and/or endangered species. Considering Enshi Tujia and Miao Autonomous Prefecture being a region known for natural production of high-quality *Dang Gui* (*Angelicae Sinensis Radix*, ASR) and *Dang Shen* (*Codonopsis Pilosula*, CP), we chose these two specific herbs for validation in further functional experimentations using AD rat models (Luo et al., 2014), among the candidate herbs (n = 13) that met the above criteria (Figure 2C). The animal experiments with rat Aβ_42_ AD models showed that rat models underwent prevention with ASR extracts demonstrated better cognitive performance in spatial probe trials, as compared with those model animals without treatment (Figure 2D; Supplementary Figure S1). Similar difference was observed between scopolamine-induced AD models subjected to prevention with CP extracts or not (Figure 2E; Supplementary Figure S1). Coherent with the behavioral phenotype, rats in ASR and CP prevention groups had higher neuron cell density in the CA1 and CA3 regions of the hippocampus as compared with the non-treated animals, as revealed by Nissl staining (Figures 2F, G; Supplementary Figure S2). We next performed RNA-seq transcriptomic profiling of the hippocampus of the ASR treated (n = 3) and non-treated (n = 3) Aβ_42_ AD rat models for a molecular understanding of its neural protection effect. GSEA revealed that the pattern of the transcriptomic difference between the hippocampus of ASR-treated versus non-treated Aβ_42_ AD rats demonstrated a reversal of the trancriptomic changes in aging brain and AD patients in humans (Lu et al., 2004; Blalock et al., 2004), suggesting possible anti-aging and anti-AD effect of ASR (Supplementary Tables S2, S4). Specifically, GSEA using single-cell signatures showed a higher expression of cell markers of various neuron cell types in the hippocampus of ASR-treated Aβ_42_ AD rats (La Manno et al., 2016). On the contrary, expression levels of vessel cell and microglia markers, which have been shown as possible pathogenic mediators in AD, were lower in the hippocampus of ASR-treated Aβ_42_ AD rats (La Manno et al., 2016; Bennett et al., 2018; d'Errico et al., 2022; Table 2). Additionally, in the hippocampus of ASR-treated Aβ_42_ AD rats, downregulation was observed of genes involved in AD-related key molecular pathways, including inflammation, IFN-response, and extracellular matrices (ECM) (Table 2; Roy et al., 2020; Johnson et al., 2023). These findings confirmed the neuroprotective effect of ASR extracts in rats, and suggested inhibiting effects on microglia, angiogenesis, inflammation, and ECM-remodeling as possible underlying mechanisms.

**TABLE 2:** GSEA of DEGs between hippocampus of Aβ_42_ AD rats subjected or not to pretreatment of ASR extracts, concerning aging and AD brain, brain cell types, and AD-related functional ontologies.

Ontologies	pval	padj	NES
LU_AGING_BRAIN_DN	1.28E-09	4.72E-07	2.33
LU_AGING_BRAIN_UP	3.29E-03	7.33E-02	−1.45
BLALOCK_ALZHEIMERS_DISEASE_DN	9.19E-07	1.42E-04	1.36
BLALOCK_ALZHEIMERS_DISEASE_UP	9.73E-04	3.24E-02	−1.26
LEIN_NEURON_MARKERS	3.60E-07	6.39E-05	2.35
MANNO_MIDBRAIN_NEUROTYPES_HGABA	3.59E-41	1.18E-36	2.34
MANNO_MIDBRAIN_NEUROTYPES_HDA1	2.72E-29	4.47E-25	2.52
MANNO_MIDBRAIN_NEUROTYPES_HNBGABA	1.92E-28	1.57E-24	2.39
MANNO_MIDBRAIN_NEUROTYPES_HDA2	1.13E-25	6.17E-22	2.51
MANNO_MIDBRAIN_NEUROTYPES_HSERT	2.80E-25	1.31E-21	2.58
MANNO_MIDBRAIN_NEUROTYPES_HNBML5	1.74E-23	5.71E-20	2.53
MANNO_MIDBRAIN_NEUROTYPES_HDA	6.28E-22	1.47E-18	2.39
MANNO_MIDBRAIN_NEUROTYPES_HOMTN	7.96E-19	1.37E-15	2.40
MANNO_MIDBRAIN_NEUROTYPES_HENDO	5.86E-18	9.16E-15	−1.89
MANNO_MIDBRAIN_NEUROTYPES_HRN	1.76E-16	2.14E-13	2.42
MANNO_MIDBRAIN_NEUROTYPES_HPERIC	4.05E-14	3.59E-11	−1.82
MANNO_MIDBRAIN_NEUROTYPES_HNBML1	5.44E-13	4.15E-10	2.24
MANNO_MIDBRAIN_NEUROTYPES_HMGL	3.96E-09	1.21E-06	−1.72
MANNO_MIDBRAIN_NEUROTYPES_HNBM	8.12E-08	1.74E-05	1.94
REACTOME_ARACHIDONIC_ACID_METABOLISM	3.67E-04	1.63E-02	−1.82
GOBP_INFLAMMATORY_RESPONSE	3.27E-05	2.64E-03	−1.51
BROWNE_INTERFERON_RESPONSIVE_GENES	1.30E-06	1.90E-04	−2.02
HALLMARK_INTERFERON_ALPHA_RESPONSE	3.60E-06	4.53E-04	−1.90
HALLMARK_INTERFERON_GAMMA_RESPONSE	1.85E-05	1.68E-03	−1.73
REACTOME_INTERFERON_ALPHA_BETA_SIGNALING	8.68E-04	3.00E-02	−1.74
NABA_MATRISOME	1.35E-17	1.92E-14	−1.96

The present case study of applying SysNatMed to AD has certain limitations that can be addressed in the future. First, regarding the *in vivo* animal experiments, we used two different AD models given that these two models are both frequently used and established AD models. Observing effectiveness in two different AD models would strengthen the validity of our computational method. Nevertheless, the Aβ_42_ and scopolamine rat models may not fully replicate the complexity of human Alzheimer’s disease, but overall these models mainly focus on amyloid accumulation and acute cognitive deficits and may lack information on the long-term effects of the treatments or their effectiveness in preventing neurodegeneration, which can be further examined in the future with other models, such as the APPswe/PSEN1dE genetically engineered mouse model. Second, earlier molecular analysis could guide the experimental design, making the study’s findings more directly aligned with the identified molecular mechanisms. For example, the identification of immune-related pathways in the therapeutic effects of ASR suggests a key role for inflammation in AD. This insight could be used in further investigations to direct immunohistochemical analyses in the rat models, allowing for a deeper exploration of microglial or astrocytic activation, a mechanism that is crucial in AD pathology. Third, same molecular analyses for CP can be done in future studies to provide biological insights of the therapeutic potential of CP for AD prevention.

Additionally, integrating other methods, such as network pharmacology, molecular docking, molecular dynamic (MD) simulation, and ADMET analysis, would be an important future direction. These methods could further enhance our biological interpretation and facilitate therapeutic translation of our findings. For example, network pharmacology could help identify potential pathway-level mechanisms by mapping ASR and CP targets onto AD-related interaction networks. Molecular docking and MD simulation could provide structural insights into the binding affinities of key bioactive compounds with AD-relevant proteins, while ADMET analysis could help prioritize compounds with favorable pharmacokinetic and safety profiles. Furthermore, integrating artificial intelligence (AI)-based methods, such as large language models (LLMs) and AlphaFold, could further expand the potential of our approach in multiple aspects, including biological interpretation and therapeutic translation. For example, LLMs could be applied to systematically analyze RNA-seq results, assist in gene-disease association extraction, and optimize biomarker discovery pipelines. Meanwhile, AlphaFold’s structural predictions could provide insights into the binding interactions between ASR/CP active compounds and key AD-related proteins, which may help refine drug-target prioritization strategies. In the present study, we focused on a systems pharmacology approach integrating genomic data and network-based analyses. While AI-powered methods are beyond the current scope, we acknowledge their transformative potential and plan to incorporate them in future research. As AI models continue to evolve, their integration into traditional medicine-based drug discovery could significantly enhance predictive accuracy and therapeutic applicability.

In summary, we developed a novel data-driven framework for rational discovery of natural medicine leveraging systems genetics, the effectiveness of which was shown in a case study using AD animal models. Our approach is transparent and cost-effective, and the required data and software tools are freely accessible to the research community. Our approach is potentially applicable to a large number of diseases, especially those defined by complex genetics and lack effective therapeutic strategies. We believe it can be a good starting point for rational exploitation of complementary medicine for human well-being.

## Data Availability

The data presented in the study are deposited in the ZENODO repository, accession number 14915194 at https://zenodo.org/records/14915194.
