# Supplementary material for: SysNatMed: rational natural medicine discovery by systems genetics
Source: Front Pharmacol. 2025 Mar 3;16:1496061. doi: 10.3389/fphar.2025.1496061 (PMC11911470; doi:10.3389/fphar.2025.1496061)
Supplement: Supplementary file 1 [file DataSheet1.pdf]

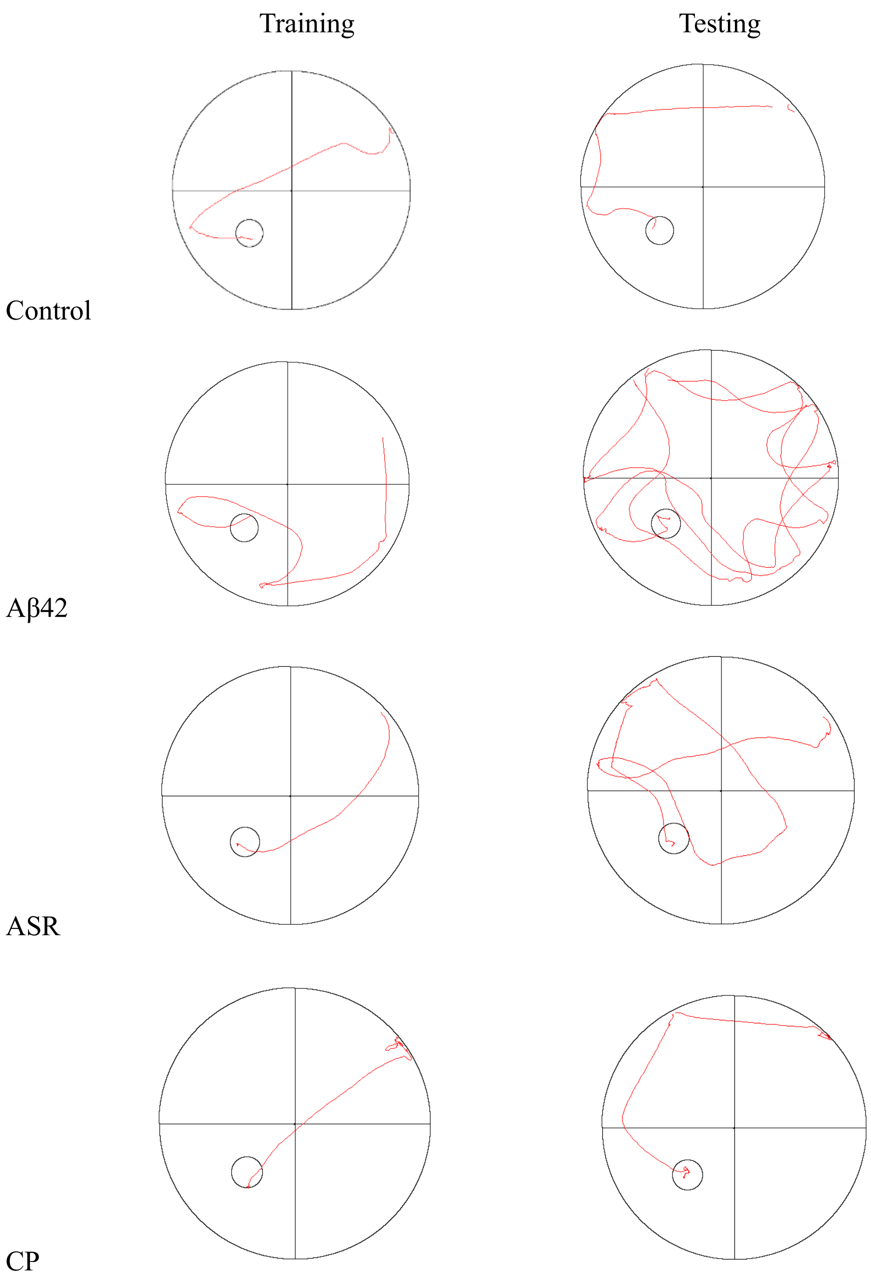

Figure S1. Representative images of swimming tracks of model animals in Morris water maze experiments in training and testing assays.

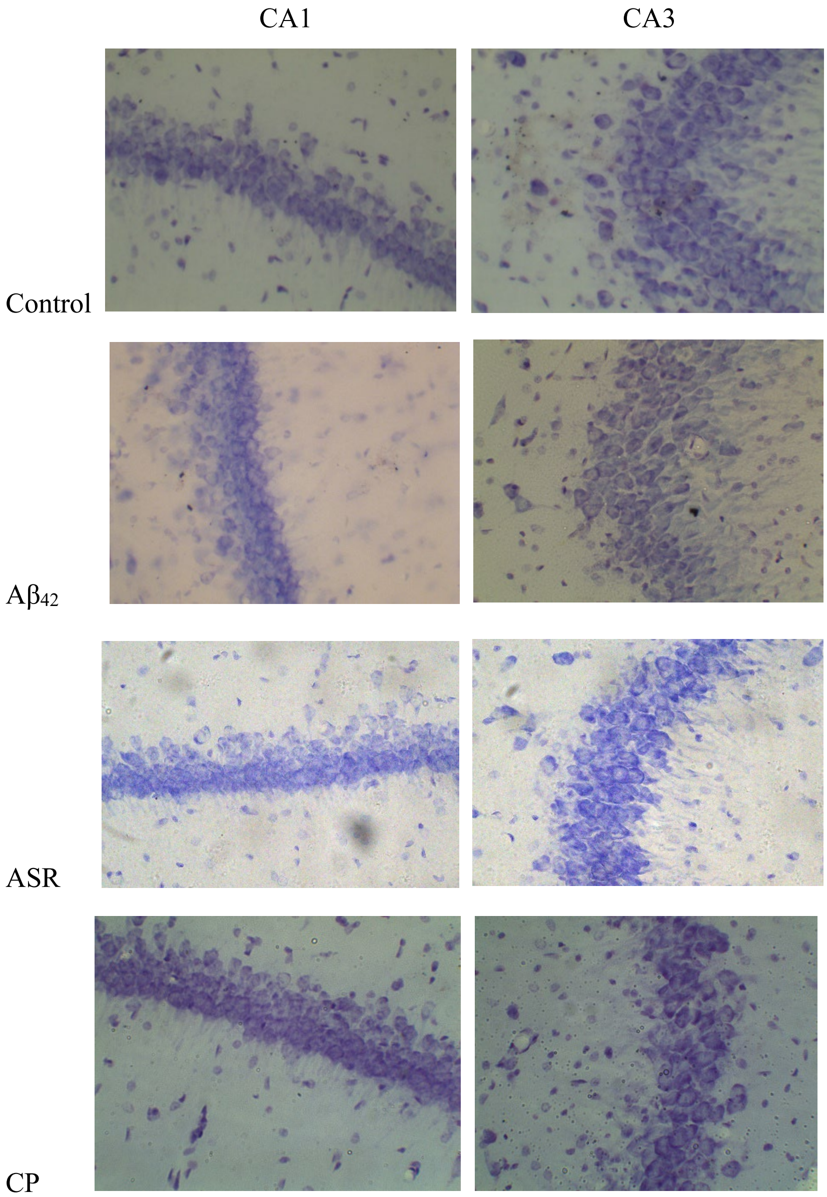

Figure S2. Representative Nissl staining images of hippocampus of rat model animals.
